# Genome wide analysis of *Arabidopsis *core promoters

**DOI:** 10.1186/1471-2164-6-25

**Published:** 2005-02-25

**Authors:** Carlos Molina, Erich Grotewold

**Affiliations:** 1 Department of Plant Cellular and Molecular Biology and Plant Biotechnology Center, The Ohio State University, Columbus, OH 43210; 2 Departamento de Informática, Universidad Técnica Federico Santa María, Valparaíso, Chile

## Abstract

**Background:**

Core promoters are the gene regulatory regions most proximal to the transcription start site (TSS), central to the formation of pre-initiation complexes and for combinatorial gene regulation. The DNA elements required for core promoter function in plants are poorly understood. To establish the sequence motifs that characterize plant core promoters and to compare them to the corresponding sequences in animals, we took advantage of available full-length cDNAs (FL-cDNAs) and predicted upstream regulatory sequences to carry out the analysis of 12,749 *Arabidopsis *core promoters.

**Results:**

Using a combination of expectation maximization and Gibbs sampling methods, we identified several motifs overrepresented in *Arabidopsis *core promoters. One of them corresponded to the TATA element, for which an in-depth analysis resulted in the generation of robust TATA Nucleotide Frequency Matrices (NFMs) capable of predicting *Arabidopsis *TATA elements with a high degree of confidence. We established that approximately 29% of all *Arabidopsis *promoters contain TATA motifs, clustered around position -32 with respect to the TSS. The presence of TATA elements was associated with genes represented more frequently in EST collections and with shorter 5' UTRs. No *cis*-elements were found over-represented in TATA-less, compared to TATA-containing promoters.

**Conclusion:**

Our studies provide a first genome-wide illustration of the composition and structure of core *Arabidopsis *promoters. The percentage of TATA-containing promoters is much lower than commonly recognized, yet comparable to the number of *Drosophila *promoters containing a TATA element. Although several other DNA elements were identified as over-represented in *Arabidopsis *promoters, they are present in only a small fraction of the genes and they represent elements not previously described in animals, suggesting a distinct architecture of the core promoters of plant and animal genes.

## Background

In eukaryotes, many cellular processes are regulated at the level of transcription. Initiation of transcription by RNA polymerase II requires the assembly of the basal transcription apparatus at the core promoter, a region of about 70 bp flanking the transcription start site (TSS) [1]. Interactions mediated by components of the basal machinery and transcription factors that recognize specific *cis*-regulatory elements, frequently located upstream of the core promoter, ensure efficient and regulated transcription by RNA polymerase II at Class II promoters [2]. Class II core promoters often contain conserved DNA elements recognized by components of the basal transcription machinery, the general transcription factors. The best-described core promoter DNA element is the TATA box, which is recognized by TATA-binding protein (TBP). The TATA box is a T/A-rich sequence usually located 25–35 base pairs upstream of the TSS [3]. Recruitment of TBP and TBP-associated factors, all part of the TFIID complex, directs assembly of the pre-initiation complex (PIC), a highly regulated process that ensures precise initiation of transcription. The directionality of the PIC is likely to be provided by the presence of another conserved element, present in a large fraction of Class II promoters, the BRE (IIB recognition element) [4,5]. In addition, Initiator (Inr) elements are often present at the site of initiation of transcription in a number of eukaryotic core promoters. The Inr is a loosely conserved element containing an adenosine at the TSS and a C as the nucleotide preceding it (position -1), surrounded by a few pyrimidines [2]. The function of the Inr and the components of the basal transcription machinery that recognize this element remain poorly defined.

In spite of the availability of a large number of computational programs that predict the presence of plant genes and their architecture (reviewed in [6]), accurately identifying core promoters solely based on genome sequence analysis remains a daunting task. Although no known DNA-sequence motif is present in all plant core promoters, TATA and Inr motifs represent two elements that are often present [7]. A main limitation in the analysis of plant core promoters is the insufficient amount of information available regarding TSSs, and hence the location of core promoters in genomic sequences. Over the past few years, several efforts have initiated the high-throughput production and analysis of full-length (FL) *Arabidopsis *cDNAs [8,9]. These FL-cDNAs have dramatically improved the annotation of the *Arabidopsis *genome [10], providing a powerful tool for the identification and analysis of core promoter elements.

Here, we describe the analysis of core promoters of *ca*. 12,750 *Arabidopsis *genes, using publicly available FL-cDNA sequences. Our objectives for this study were to i) identify motifs characteristic of *Arabidopsis *core promoters; ii) determine how often *Arabidopsis *core promoters contain a TATA box, and iii) compare the architecture of *Arabidopsis *core promoters with those of *Drosophila*, the only higher eukaryote for which such a genome-wide analysis has been performed. We examined the presence, distribution and consensus sequence of conserved motifs proximal to the TSS. In addition to TATA elements, we identified several other motifs, primarily representing microsatellite elements, some of them overrepresented in particular regions of core promoters. Using Nucleotide Frequency Matrices (NFM), we carried out a genome-wide analysis for the presence and position of TATA-box elements. Our studies show that only about 29% of all *Arabidopsis *genes contain a recognizable TATA element. The position of the TATA motif with respect to the TSS and correlations between the presence of a TATA with EST abundance and 5' UTR lengths are discussed.

## Results and discussion

### Obtaining core promoter and 5' UTR sequences for 12,749 *Arabidopsis *genes

As a first step towards identifying core *Arabidopsis *promoters, we queried TAIR's Gene Search with the condition of a FL-cDNA entry. We retrieved a total of 13,964 non-redundant hits, derived from over 28,000 total FL-cDNAs deposited at TAIR. The locus Ids for these 13,964 FL-cDNAs was used to retrieve the 5' UTR corresponding to 12,749 genes. The remaining 1,215 genes for which a 5' UTR was not retrieved corresponded to FL-cDNAs that differed between the annotations at TAIR and TIGR, sequences for which no 5'UTR was annotated or sequences with 5' UTR regions corresponding to alternative gene models.

The [-500, -1] and [-50, -1] regions of all 12,749 genes was directly retrieved from the TAIR 500 bp upstream dataset. To obtain the [+1, +50] regions, we first checked the length of the 5' UTRs, which was shorter than 50 bp for 2,649 genes and which was interrupted by introns in 2,179 genes. To include into our analyses these cases, three different strategies were followed. If the 5' UTR was longer than 50 bp, and no introns were present in the corresponding [-50, -1] region (10,100 genes), a direct retrieval of the [+1, +50] region was performed from the TAIR 5' UTR dataset. If the 5' UTR was shorter than 50 bp and no intron interrupted this region (2,617 genes), we extended the 5' UTR to 50 nt with a fragment of the immediately adjacent downstream coding sequence using the TIGR cDNA dataset. Finally, if the 5' UTR was shorter than 50 nt and an intron interrupted this region (32 genes), we manually retrieved the [+1, +50] region from the genomic sequence using TAIR's SeqViewer. After these analyzes, we were able to generate datasets corresponding to the [-500, -1], [-50, -1] and [+1, +50] regions from a total of 12,749 genes. These datasets were used for all the subsequent analyzes in this study.

### Identification of conserved motifs in core promoters

**Figure 1:**
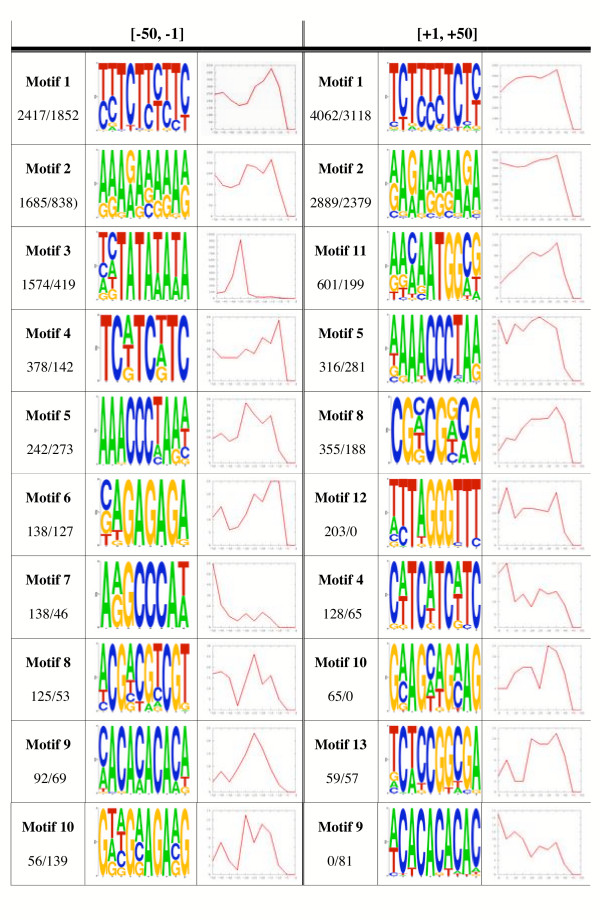
Analysis of motifs present in the [-50, -1] and [+1, +50] regions of 12,749 *Arabidopsis *genes. Motifs are numbered from 1 to 13 and ordered by the number of occurrences, indicated by the numbers under the motif name. The first numeral corresponds to the number of hits using MEME, the second to the number of hits using AlignACE. For example, 2417/1852 indicates a motif found 2,417 times using MEME and 1,852 times with AlignACE. The second column for each motif shows the nucleotide frequency distribution graphed using WebLogo, where the sizes of the characters represent the frequencies of occurrence. The third column provides a graphic representation of the frequency distribution (y-axis) of each motif in the [-50, -1] or [+1, +50] regions (x-axis).

To identify sequence motifs overrepresented in *Arabidopsis *core promoters, we first searched for DNA elements conserved in the [-50, -1] and [+1, +50] regions of the 12,749 *Arabidopsis *genes. The search was carried using both MEME and AlignACE (see Methods). Motifs correspond to short sequences (6–10 bp), often recognized by a DNA-binding protein, and which can be represented by a consensus sequence. While the total number of motifs retrieved per region with these algorithms was 16 and 32 respectively, only motifs detected in at least 50 sequences with either MEME or AlignACE are shown (Figure 1). A comprehensive list and sequence of the remaining motifs is provided as Additional File 1. From 20 motifs present in 50 or more sequences in the [-50, -1] or [+1, +50] regions, seven were present in both regions (Motifs 1, 2, 4, 5, 8, 9 and 10; Figure 1), and thus were given the same numbers. Motifs 5 and 12 are reverse-complements of each other, and they are shown separately because they are over-represented in different regions of the core-promoters (Figure 1). Overall, the expectation maximization method MEME appears to be a more robust motif search algorithm than the Gibbs sampling method, AlignACE, since MEME resulted in a significant higher rate of identification for most of the motifs (Figure 1). Two motifs identified by MEME (Motifs 10 and 12, Figure 1) were not identified by AlignACE in any significant number of sequences. The distribution of the different motifs within the [-50, -1] or [+1, +50] regions was also investigated (Figure 1). In a few cases, there was a clear enrichment of motifs at particular positions. For example, Motif 3, only present in the [-50, -1] region, was clustered in the -30 to -45 region, Motif 9, present in both regions, clustered closer to the TSS and Motif 7 showed an enrichment in the vicinity of the -50 position (Figure 1).

### Overrepresentation of motifs in the [-50, -1] or [+1, +50] regions

To investigate whether the number of sequences containing each one of these motifs was accurately predicted by MEME or AlignACE and to establish which of these 13 motifs was significantly overrepresented in the [-50, -1] or [+1, +50] regions, we retrieved nucleotide frequency matrices (NFMs) for each one of these motifs from the results of the MEME search (see Methods). The NFMs for each of these motifs, provided as Additional File 2, were used to determine their presence in the [-50, -1] or [+1, +50] regions. To establish whether the motifs were overrepresented in these regions, we used two background models. The first background model corresponded to an identical number of random sequences (columns 4 and 6 in Table 1 labeled Random) with the same nucleotide composition as the [-50, -1] or [+1, +50] regions. Because biological sequences are not random and intragenic sequences are richer in homopolymeric A/T than predicted by a random model with identical nucleotide composition, we used as the second background model the 12,749 non-core promoter [-500, -450] regions. The results are shown in Table 1 (column 2 in Table 1 labeled Real).

**Table 1:** Motif frequency in the [-50, -1] and [+1, +50] regions of 12,749 *Arabidopsis *genes compared to background models

	**[-500, -450]**	**[-50, -1]**		**[+1, +50]**	
	**Real**	**Real**	**Random**	Real*	**Random**

**Motif 1**	1388	3817	778	4379 (3769)	806
**Motif 2**	1558	2195	235	3018 (2312)	323
**Motif 3**	543	1899	289	314 (289)	243
**Motif 4**	241	1288	109	1665 (1427)	114
**Motif 5**	88	382	49	421 (361)	54
**Motif 6**	275	477	56	894 (690)	81
**Motif 7**	59	153	34	28 (27)	51
**Motif 8**	157	282	83	421 (279)	106
**Motif 9**	208	385	111	519 (416)	163
**Motif 10**	168	270	21	519 (340)	23
**Motif 11**	548	460	297	1352 (213)	362
**Motif 12**	137	253	57	346 (308)	61
**Motif 13**	175	183	113	343 (241)	111

*Number in parentheses indicate the frequency of the motif in the 10,100 [+1, +50] 5' UTR sequences without introns or coding regions.

Motifs 3 and Motifs 7 showed a clear Overrepresentation in the [-50, -1] interval. Motif 3 has all the characteristics of a TATA box (Figure 1), and was detected in 1,899 genes using the NFM, representing approximately 15% of all the genes investigated. A more detailed characterization of this motif is described below. Motif 7 was detected in a much smaller number of genes (153), and the corresponding motif with the A A/G GCCCA T/A consensus was shown before to be overrepresented in upstream regions versus coding regions of *Arabidopsis *genes [11]. Consistent with our findings that show an increased accumulation of this motif towards the left border of the [-50, -1] interval (Figure 1), this motif was previously shown to have a strong positional preference for the [-250, -50] interval [11]. Interestingly, in *Arabidopsis *this motif is associated with dark-induced genes and is over-represented in genes under circadian regulation [12].

Three motifs were also found to be overrepresented in the [+1, +50] region. Motif 10 resembles the (GAA)_n _microsatellite represented at least two fold higher in the [+1, +50] region, compared to the [-50, -1] or the [-500, -450] regions (Table 1). This overrepresentation cannot be explained by the modest difference in nucleotide composition between these regions, consistent with the comparable distribution in the randomly simulated datasets (Table 1). As described above, 2,649 of the [+1, +50] regions contain coding regions in addition to short 5' UTRs. To investigate whether the coding sequences contributed to the overrepresentation of this motif, we analyzed the presence of this motif in the 10,100 [+1, +50] "clean" 5'UTR regions, which do not contain any coding or intron sequences (shown between brackets in Table 1 under [+1, +50] Real). In these 10,100 sequences, Motif 10 was found in 340 [+1, +50] sequences, the same frequency as in the original dataset (519/12,749). Thus, this (GAA)_n _microsatellite is overrepresented in the [+50, +1] region, irrespective of whether it is coding or 5' UTR. (GAA)_n _microsatellites have been extensively researched in humans [13], but not yet associated with any functional role in *Arabidopsis*.

Motif 13, with the consensus T/A CCGGCGA (Figure 1), was detected by both MEME and AlignACE only in the [+1, +50] region (Table 1). This motif, however, was not identified as the binding site for any known transcription factor, as deduced from searching the PLACE [14], TRANSFAC [15] and AGRIS [16] databases (not shown).

Finally, Motif 11, present in a significant number of sequences (Figure 1), fits the Kozak consensus (ACCATGG) for a translation start ATG codon [17]. Consistently, 1,139 out of the 1,352 sequences in which we found Motif 11 have a short 5' UTR, reflected in that this motif is present in just 213 5' UTR [+1, +50] sequences (Table 1). While this motif is irrelevant to our analysis, it provides a good internal control regarding the sensitivity and comprehensiveness of our search for motifs in the [-50, -1] and [+1, +50] regions.

Motifs 1, 2, 4, 6, and 9 correspond to microsatellites commonly found in *Arabidopsis *[18], displaying similar frequency distributions in the [-50, -1] and [+1, +50] regions. From these 5 motifs, only Motif 2 does not seem to be significantly overrepresented in these two regions, when compared to the [-500, -450] sequences (Table 1). The potential participation of microsatellites in the control of gene expression is unclear, but according to recent studies in rice and *Arabidopsis*, their distribution may follow a gradient in the direction of transcription [18]. Motif 8 conforms to a (CG)_n _microsatellite, frequent in monocots such as rice, but not often found in *Arabidopsis *[18], which is consistent with a low but comparable frequency in all three regions studied here (Table 1). The apparent higher frequency of Motif 8 in the [+1, +50] region, compared to the [-50, -1] (421 versus 282, respectively), is likely to correspond to an increased G/C content of the 5' UTR (see Methods), as reflected by the increased distribution of this motif in a random simulation of sequences with the same nucleotide composition of the corresponding [+1, +50] region (Table 1). Motif 9, corresponding to a (CA)_n _microsatellite (with n = 5), was found to be only slightly overrepresented in the [-50, -1] region, compared to the [-500, -450] background model (Table 1). Interestingly, however, this motif is significantly clustered in the [-35, -10] region (Figure 1). A similar clustering was not observed in the [+1, +50] region, where this motif is significantly overrepresented, compared to the background models (Table 1).

Motif 5, with the consensus sequence AAACCCTA (Fig. 1), and similarly overrepresented in the [-50, -1] and [+1, +50] regions, compared to the random or [-500, -450] background models (Table 1), does not conform to a typical microsatellite sequence. Interestingly, however, the sequence of Motif 5 is precisely the reverse complement of Motif 12, which with the TAGGGTTT DNA-consensus fits the sequence of the *Arabidopsis *telomeric sequence [19], and of the telobox, the binding site for a MYB-related telomeric DNA-binding protein previously described in proteins from yeast, plants and animals [20]. This element, present in the 5' UTR or promoter region of many genes encoding products associated with the translational apparatus [21], was also shown to participate in the expression of *Arabidopsis *root meristem genes [22]. Our analysis suggests that the number of sequences containing the telobox motif in either the forward or reverse-complement configuration is much larger than previously reported [23]. Consistent with previous studies [23], only a few genes (8) contain Motif 5 or 12 in both the [-50, -1] and [+1, +50] regions.

We also investigated for the presence of motifs previously shown to be overrepresented in the [-60, +40] regions of *Drosophila *core promoters [24]. Using the corresponding NFMs, we searched our databases for DRE (DNA-replication related element) and DPE (downstream promoter element), usually found ~30 nt downstream of the TSS [25,26]. Although the [-60, +40] region is shifted 10 bp towards the 5' end from our selection, the positional clustering of the DRE and DPE motifs [24] still falls under the [-50, +50] region investigated here. In our analyses, neither one of the two motifs was represented at a level significantly higher than in the random models (not shown). A CCAAT box NFM [7] did not result in any significant distribution change between real and randomly generated datasets for both regions (not shown). This was expected because CCAAT boxes usually cluster around the -75 position [27], which is outside of the [-50, +50] interval investigated here, corresponding to what is generally recognized as the core promoter region. Similarly, none of the motifs identified here appeared to correspond to Inr elements. We conclude that, with the exception of the TATA box, the elements involved in the architecture of core promoter in *Arabidopsis *and *Drosophila *are overall different.

### Distribution of TATA motifs in core *Arabidopsis *promoters

**Figure 2:**
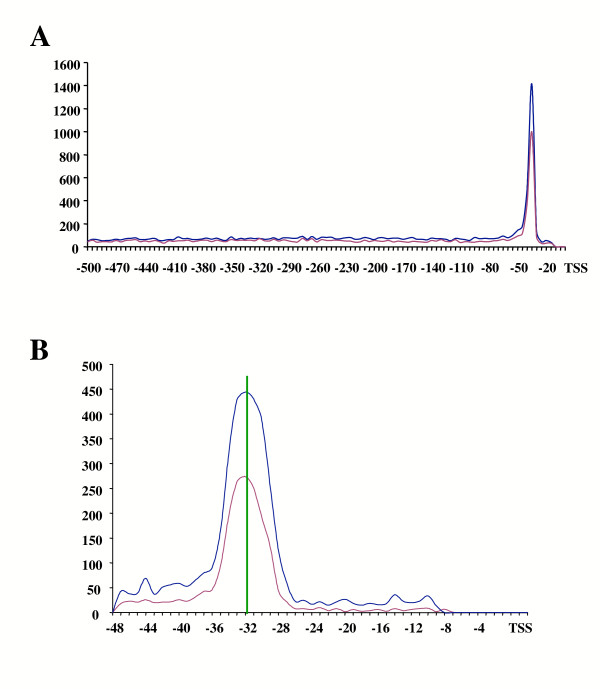
Position of TATA motifs in *Arabidopsis *promoters. A, The analysis of the 12,749 [-500, -1] regions with the MEME-derived NFM (Table 3) resulted in 6,316 sequences containing a significant hit (indicated by the red curve), 1,768 of them clustered in the [-50, -1] region. A similar analysis with the expanded and improved PlantProm-derived NFM (Table 4) resulted in 8,776 hits (blue curve), 2,507 of them clustered in the [-50, -1] region. B, Expansion of the [-50, -1] region indicating with a vertical green line that the average distance of the TATA motifs present in the [-50, -1] region is 31.7 nt from the TSS (using the first conserved T as the reference position).

According to our analysis for conserved core promoter elements, Motif 3 (Figure 1) is likely to represent the TATA box characteristic of many Class II promoters. Consistent with this idea, Motif 3 is significantly overrepresented in the [-50, -1] region (Table 1) with a clear clustering in the -30 to -45 region (Figure 1). Surprisingly, however, Motif 3 was only detected in 15% of the 12,749 core promoters investigated, lower than found in previous studies, which suggested that 57% of plant genes had a TATA box [7]. To investigate this striking difference between previous estimates for the frequency of a TATA box in *Arabidopsis *promoters and our own analyses, we utilized the previously described TATA NFM [7]. With this NFM, MotifScanner identified 3,679 TATA motifs in the [-50, -1] region, significantly higher than the number of hits in the [+1, +50] region, or in the corresponding background models (Table 1). Thus, according to this analysis, 28.8% of all *Arabidopsis *genes contain a TATA, comparable to the number of *Drosophila *core promoters suggested to contain a TATA box (28–34%) [24], but still significantly lower than previously reported for the analysis of 305 plant promoters [7]. Interestingly, however, if these prior studies are restricted to just the 63 sequences from *Arabidopsis*, only 23 showed the presence of a TATA, representing a frequency of 36.5%, comparable to our own results. Previous studies also suggested that plant TATA-less promoter were the exception [28], and that TATA-less promoters were mainly restricted to photosynthetic [28] and plastid ribosomal genes [29]. Our results, however, indicate that TATA-less promoters are found more frequently than TATA-containing promoters. We cannot rule out that *Arabidopsis *is the exception among the plants, a possibility to be considered given the much lower percentage of TATA-containing promoters in *Arabidopsis *compared to other plants [7]. More likely, however, the lack of a good knowledge of the position of the TSS may have resulted in previous studies in a very significant over-estimate of the presence of TATA elements. As an example, if the search for TATA elements is carried out on the 12,749 [-500, -1] regions, 6,316 sequences (using the MEME NFM) or 8,776 (using the expanded PlantProm NFM) are retrieved as containing a significant hit to a TATA element (Figure 2A), corresponding to 49.5% and 70% respectively, much closer to previous, yet likely incorrect, estimates [28].

The sequences from all these putative TATA-containing promoters were retrieved and the NFMs were retrained with this new information. The new matrix obtained from 1,899 sequences gathered using our MEME NFM (Figure 1) is shown in Table 3. Similarly, the PlantProm TATA NFM was retrained with the 3,679 sequences, resulting in an improved and expanded NFM (Table 4). These NFMs provide robust tools for the identification of additional plant TATA motifs. The two NFMs are significantly better than previously available plant TATA NFMs, with regards to the addition of flanking sequences that permit to expand the TATA consensus, and because of the much larger number of sequences used to build them. They have very similar nucleotide distributions, probably the biggest difference being at position 8, were the matrix derived from our MEME analysis has a much stronger requirement for an A (compare Tables 3 and 4).

**Table 3:** TATA NFM derived from 1,899 motifs.

**Derived from MEME**																
	**-4**	**-3**	**-2**	**-1**	**1**	**2**	**3**	**4**	**5**	**6**	**7**	**8**	**9**	**10**	**11**	**12**

**A**	0.227	0.259	0.244	0.245	0.003	0.997	0.001	0.994	0.408	0.994	0.358	0.906	0.241	0.439	0.302	0.393
**C**	0.244	0.262	0.230	0.398	0.001	0.001	0.002	0.003	0.001	0.001	0.001	0.003	0.294	0.228	0.269	0.204
**G**	0.125	0.180	0.113	0.153	0.002	0.001	0.000	0.001	0.001	0.002	0.001	0.090	0.193	0.153	0.160	0.161
**T**	0.403	0.300	0.413	0.203	0.994	0.001	0.997	0.002	0.590	0.003	0.641	0.002	0.272	0.180	0.270	0.242
	**t**	**n**	**t**	**c**	**T**	**A**	**T**	**A**	**T/A**	**A**	**T/A**	**A**	**n**	**a**	**n**	**a**

**Table 4:** TATA NFM derived from 3,679 motifs.

**Derived from PlantProm**																
	**-4**	**-3**	**-2**	**-1**	**1**	**2**	**3**	**4**	**5**	**6**	**7**	**8**	**9**	**10**	**11**	**12**

**A**	0.246	0.262	0.248	0.243	0.058	0.917	0.000	0.998	0.493	0.943	0.417	0.655	0.197	0.399	0.340	0.383
**C**	0.246	0.247	0.242	0.434	0.030	0.000	0.049	0.001	0.000	0.001	0.020	0.093	0.349	0.286	0.244	0.212
**G**	0.118	0.184	0.126	0.111	0.000	0.001	0.000	0.000	0.000	0.038	0.000	0.100	0.221	0.159	0.141	0.141
**T**	0.391	0.308	0.384	0.213	0.911	0.083	0.951	0.001	0.507	0.018	0.563	0.153	0.232	0.156	0.275	0.264
	**t**	**n**	**t**	**c**	**T**	**A**	**T**	**A**	**T/A**	**A**	**T/A**	**A**	**c**	**a**	**a**	**a**

The new NFMs were used to scan the [-500, -1] region and establish where each of them localized a TATA with the highest probability. As shown in Figure 2A, both NFMs showed a significant peak in the [-50, -25] region, consistent with the position expected for TATA elements. To establish the average distance of TATA elements to the TSS, the MEME and PlantProm TATA NFMs were used to scan the 12,749 [-50, -1] regions and the positions of the corresponding TATA boxes were recorded and graphed (Figure 2B). The average distance of a TATA (position 1 in Tables 3 and 4) to the TSS is 31.7 nt (indicated with a green line in Figure 2B). Thus, the position of the TATA box in *Arabidopsis *is more similar to what is typically the case in animal promoters, usually 25–30 nt from the TSS [2] than what is found in yeast, where the TATA box has a variable position in the [-100, -40] region [30].

**Figure 3:**
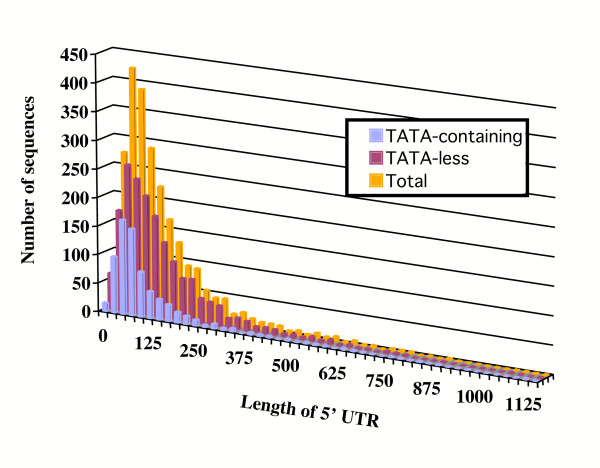
Length distribution of 5' UTRs in TATA-containing and TATA-less genes. The length of the 5' UTR of all 12,749 genes (orange bars) shows an average of 129 nt. Promoters lacking a TATA box (TATA-less, red bars) have in average 5' UTRs 138 nt long. The 5' UTR of TATA-containing genes (blue bars) are in average 108 nt long.

We investigated whether the presence of TATA motifs correlated with other properties of the corresponding genes. Based on our analysis of the 12,749 FL-cDNAs, we determined that the average size of the 5' UTR of *Arabidopsis *genes is 129 nt (Figure 3). Interestingly, when we compared the average length of the 5' UTRs of TATA-containing versus TATA-less genes, we found that TATA-containing genes had an average of 108 nt in their 5' UTRs, compared to 138 nt in TATA-less genes. This difference in the length of the 5' UTRs between these three populations of genes is evident in the sway towards shorter 5' UTRs in the TATA-containing population (Figure 3). The reason for this difference in 5' UTR length between TATA-containing and TATA-less promoter is not clear, although it is possible that the longer 5' UTR provide additional features that contribute to PIC assembly. We also investigated whether the presence of a TATA element made a difference in the times that each gene was represented in ESTs, an approximate indication of the relative level of expression of the corresponding gene. While each *Arabidopsis *gene is represented in average by 9.48 ESTs (see Methods), the 12,749 sequences utilized here are represented in average by 13.02 ESTs, suggesting that the available FL-cDNAs are likely to correspond to genes expressed at a higher level than the average *Arabidopsis *gene. Interestingly, however, TATA-containing genes were represented in average by 17.6 ESTs (17.68 using the MEME NFM and 17.52 using the PlantProm NFM, Tables 3 and 4), whereas TATA-less genes were represented by just 11.23 ESTs. These results suggest that the presence of a TATA is generally associated with genes expressed at a higher level. Gene Ontology analyses (see Methods) did not provide any insights on possible cellular functions associated with these gene clusters (not shown). An analysis of the sequences flanking the TSS, and likely containing the Inr element, did not reveal any significant difference in nucleotide composition between TATA-containing and TATA-less promoters (data not shown). Thus, the assembly of the PIC is likely to occur in *Arabidopsis *TATA-less promoters solely through the Inr, or regulatory elements outside of the [-50, +50] region investigated here also participate in the recognition of the core promoter by components of the basal transcriptional machinery.

## Conclusion

Understanding the architecture of core promoters is central to establishing the mechanisms by which the basal transcriptional machinery assembles and facilitates formation of the pre-initiation complex. We provide here the first genome-wide analysis of *Arabidopsis *core promoters. We have identified several motifs overrepresented in core promoters, with respect to background models consisting of random sequences of identical nucleotide composition or intergenic regions. With the exception of microsatellites similarly distributed in the [-50, +1] and [+1, +50] regions and the TATA element, for which an in-depth analysis was carried out, most other overrepresented motifs were present in only a small subset of the sequences analyzed. Our studies provide robust NFMs corresponding to TATA elements and other conserved motifs, and show that only 29% of all *Arabidopsis *promoters contain a TATA element located in average approximately 32 nt upstream of the TSS. The absence of a TATA correlates with a lower representation of the corresponding gene in public EST collections as well as with longer 5' UTR sequences. However, the absence of a TATA is not compensated for by the overrepresentation of any one of several motifs present in *Drosophila *core promoters, suggesting significant differences in the organization of core promoters from animals and plants.

## Methods

### Retrieval of core promoter and 5' UTR sequences

To obtain the sequences of the region of promoters spanning the first 500 nt upstream of the TSS [-500, -1] and the corresponding 5' UTRs, we used the TAIR Gene Search web tool [31]. The TAIR database was queried for all genes having a full-length cDNA (FL-cDNA) entry. The corresponding 5' UTR and the [-500, -1] regions datasets were downloaded from TAIR [32], last updated on February 28, 2004. The FL-cDNA sequences were obtained from the June 10, 2004 realese of the TIGR's cDNA dataset [33]. The locus Ids of the gene queries were checked against the 5' UTR, [-500, -1] and FL-cDNA files to reject erroneous annotations. We divided the 100 bp region flanking the TSS in upstream [-50, -1] and downstream [+1, +50] sub-regions of 50 bp each. The [-50, -1] and [+1, +50] intervals of the confirmed genes were directly retrieved from the downloaded TAIR files, when possible. In those cases when the 5' UTR region was shorter than 50 bp, the TIGR file was used to extend the region to the necessary length by appending a fragment of the immediately adjacent coding sequence. When an intron interrupted the 5' UTR, we manually extracted the 50 bp region from the *Arabidopsis *genomic sequences using the SeqViewer tool at TAIR.

### Motif discovery and motif search

To characterize core promoters, we first investigated features represented by conserved regions or motifs. From several algorithms available [34], we chose the expectation maximization method MEME (version 3.0.8) [35] and the Gibbs Sampling method AlignACE [36]. MEME and AlignACE were run for the [-50, -1] and [+1, +50] regions separately for the entire set of genes. For MEME, a fixed minimum motif length of 5 and a maximum of 10 was set and 20 motifs were requested using the zero or one occurrence per sequence model. For AlignACE, only the background fractional GC content of the input sequences was supplied, and all the other parameters were left at default values. MEME and AlignACE were run in the Itanium 2 Cluster at the Ohio Supercomputer Center. The results obtained with MEME were compared with those obtained with AlignACE. Motifs consisting of single nucleotide repeats (i.e. A_n_) were manually parsed out independent of the number of occurrences or positional preferences. The obtained motifs were plotted according to their positions within the regions and their consensus sequences were graphed using WebLogo version 2.7 [37].

To find pre-defined motifs in the [-50, -1] and [+1, +50] regions, we used the higher order probabilistic model MotifScanner from MotifSampler version 3.0 [38]. The searches were fed with the nucleotide frequency matrices (NFMs) of the selected motifs obtained from the MEME search, and a background model of order 1 accounting for single- and di-nucleotide distributions for each set. The prior probability of finding one instance of the motif was left to the default value of 0.2. We also ran the motif search with elements conserved in core promoters of other organisms. The first two corresponded to the TATA and CCAAT elements obtained as NFMs from PlantProm [7]. The other two corresponded to the Downstream Promoter Element (DPE) and the DNA-replication Related Element (DRE) described for *Drosophila *core promoters [24]. For these new four elements, we performed the same analysis as described before, using the [-50, -1] and [+1, +50] region datasets and the corresponding randomly generated dataset.

### Generation of random sequence models

After establishing that the distribution of nucleotides in the *Arabidopsis *[-50, -1] and [+1, +50] regions are ~65% A/T to ~35% C/G and ~61% A/T to ~39% C/G, respectively, a pseudo-random set of 50 bp sequences was generated for each region to be tested with the matrices as a way of determining the chances of finding the motifs candidates in a stochastic environment. This information was then used together with the search results obtained from the real data to support the confidence of the findings.

### Analysis of TATA elements

For the analysis of the TATA motif, the TATA NFM previously described [7] was used against the NFM reported by our own motif search. Using MotifScanner, the distribution of TATA elements in the upstream vicinity of the TSS was investigated. After determining the location of the putative TATA motifs in the [-50, -1] region, the NFMs were retrained with the new retrieved TATA motifs.

### Analysis of gene ontology and expression level based on EST abundance

To determine whether the occurrences of the discovered motifs were associated with specific gene functions or products we retrieved the Arabidopsis Gene Ontology Database [39] (last update July 20, 2004) and correlated the annotated molecular function, biological process or cellular component of *Arabidopsis *genes with the ones found in the motif clusters. Under the assumption that the contribution of a gene to transcription activity is related to the number of its detected ESTs, we downloaded a dataset from TAIR that accounts for the number of ESTs submissions per locus [40] (last update July 23, 2004). With this, we then established the relative expression levels based on the ratio of the genes containing a particular motif and the overall EST frequency per gene.

## List of abbreviations

bp, base pair; EST, expressed sequence tag; FL-cDNA, full-length cDNA; Inr, Initiator element; NFM, nucleotide frequency matrix; nt, nucleotide; PIC, pre-initiation complex; TBP, TATA-binding protein; TSS, translations start site 5' UTR, 5' untranslated region

## Authors' contributions

C.M. carried out all the analyses and interpreted the results. E.G. was involved in the design and supervision of the project. C.M. and E.G. jointly wrote the manuscript. Both authors read and approved the final manuscript.

**Table 2:** Frequency of TATA frequency in the [-50, -1] and [+1, +50] regions of 12,749 *Arabidopsis *genes compared to background models

	**[-500, -450]**	**[-50, -1]**		**[+1, +50]**	
	**Real**	**Real**	**Random**	**Real**	**Random**

**MEME**	543	1899	289	314	243
**PlantProm**	1526	3678	1431	1084	1209

## Supplementary Material

Additional File 1 Complete list of the motifs present in the [-50, -1] and [+1, +50] regions of 12,749 *Arabidopsis *genes. The analysis was carried out as described for the results shown in Figure 1. Click here for file

Additional File 2 Nucleotide Frequency Matrices for all the motifs shown in Figure 1. Click here for file
