# Supplementary material for: Genome wide analysis of Arabidopsis core promoters
Source: BMC Genomics. 2005 Feb 25;6:25. doi: 10.1186/1471-2164-6-25 (PMC554773; doi:10.1186/1471-2164-6-25)
Supplement: Additional File 1 — Complete list of the motifs present in the [-50, -1] and [+1, +50] regions of 12,749 Arabidopsis genes. The analysis was carried out as described for the results shown in Figure 1. [file 1471-2164-6-25-S1.pdf]

Complete list of the motifs present in the [-50, -1] and [+1, +50] regions

[-50,-1]		[+1,+50]	
**Motif 14** 390/0	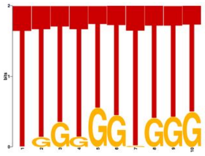	**Motif 14** 159/0	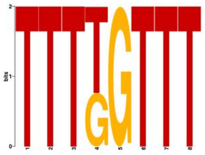
**Motif 15** 311/53	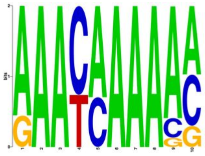	**Motif 15** 150/787	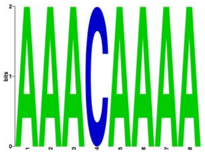
**Motif 16** 47/0	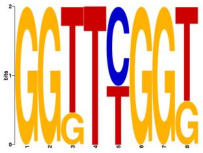	**Motif 24** 75/0	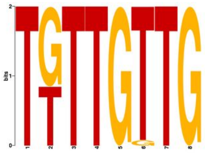
**Motif 17** 22/23	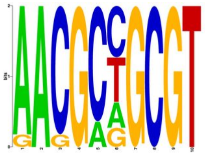	**Motif 25** 33/0	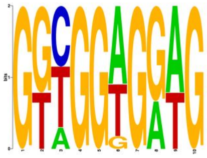
**Motif 18** 15/20	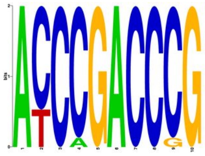	**Motif 26** 10/0	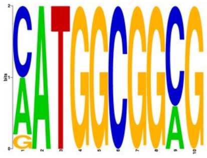
**Motif 19** 0/18	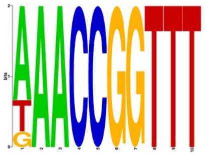	**Motif 27** 0/54	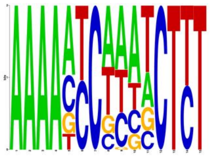
**Motif 20** 0/28	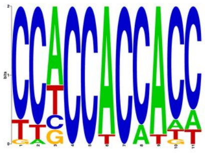	**Motif 28** 0/64	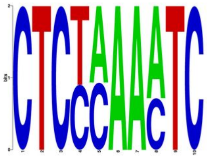
**Motif 21** 0/13	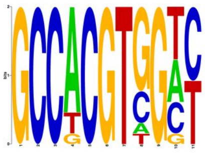	**Motif 29** 0/89	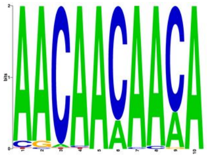
**Motif 22** 0/44	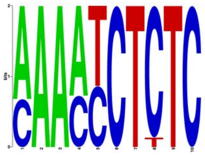	**Motif 30** 0/75	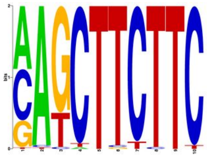
**Motif 23** 0/27	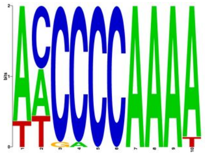	**Motif 31** 0/24	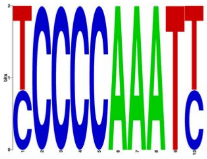
