# Supplementary material for: Genome wide analysis of Arabidopsis core promoters
Source: BMC Genomics. 2005 Feb 25;6:25. doi: 10.1186/1471-2164-6-25 (PMC554773; doi:10.1186/1471-2164-6-25)
Supplement: Additional File 2 — Nucleotide Frequency Matrices for all the motifs shown in Figure 1. [file 1471-2164-6-25-S2.pdf]

Additional File 2

NFM for all the motifs shown in Figure 1

Motif 1

A	C	G	T
0.000	0.430	0.014	0.556
0.057	0.391	0.067	0.485
0.000	0.017	0.000	0.983
0.000	0.918	0.000	0.082
0.000	0.027	0.010	0.964
0.000	0.380	0.019	0.601
0.000	0.554	0.000	0.446
0.000	0.409	0.040	0.552
0.000	0.163	0.000	0.837
0.000	0.761	0.000	0.239

Motif 2

A	C	G	T
0.784	0.000	0.216	0.000
0.686	0.000	0.314	0.000
0.920	0.000	0.080	0.000
0.497	0.000	0.503	0.000
0.830	0.000	0.170	0.000
0.558	0.212	0.230	0.000
0.744	0.000	0.256	0.000
0.659	0.000	0.341	0.000
0.935	0.000	0.065	0.000
0.517	0.000	0.483	0.000

Motif 3

A	C	G	T
0.223	0.227	0.102	0.449
0.250	0.414	0.161	0.175
0.000	0.000	0.000	1.000
1.000	0.000	0.000	0.000
0.000	0.000	0.000	1.000
1.000	0.000	0.000	0.000
0.397	0.000	0.000	0.603
1.000	0.000	0.000	0.000
0.341	0.000	0.000	0.659
0.994	0.000	0.006	0.000

Motif 4

A	C	G	T
0.000	0.000	0.000	1.000

0.000	1.000	0.000	0.000
0.447	0.000	0.212	0.341
0.000	0.000	0.000	1.000
0.000	1.000	0.000	0.000
0.339	0.000	0.222	0.439
0.000	0.000	0.000	1.000
0.000	1.000	0.000	0.000

Motif 5

A	C	G	T
1.000	0.000	0.000	0.000
1.000	0.000	0.000	0.000
0.971	0.000	0.029	0.000
0.000	1.000	0.000	0.000
0.000	1.000	0.000	0.000
0.000	1.000	0.000	0.000
0.190	0.107	0.000	0.702
1.000	0.000	0.000	0.000
0.773	0.000	0.227	0.000
0.467	0.174	0.087	0.273

Motif 6

A	C	G	T
0.000	0.471	0.355	0.174
0.768	0.000	0.080	0.152
0.000	0.000	1.000	0.000
1.000	0.000	0.000	0.000
0.000	0.000	1.000	0.000
1.000	0.000	0.000	0.000
0.000	0.000	1.000	0.000
0.906	0.000	0.094	0.000

Motif 7

A	C	G	T
1.000	0.000	0.000	0.000
0.500	0.000	0.500	0.000
0.000	0.000	1.000	0.000
0.000	1.000	0.000	0.000
0.000	1.000	0.000	0.000
0.000	1.000	0.000	0.000
1.000	0.000	0.000	0.000
0.478	0.000	0.000	0.522

Motif 8

A	C	G	T
0.680	0.128	0.008	0.184
0.000	1.000	0.000	0.000
0.000	0.000	1.000	0.000
0.536	0.136	0.136	0.192
0.000	0.856	0.000	0.144
0.112	0.000	0.888	0.000
0.080	0.216	0.064	0.640
0.000	1.000	0.000	0.000
0.000	0.000	1.000	0.000
0.016	0.000	0.112	0.872

Motif 9

A	C	G	T
0.315	0.609	0.022	0.054
0.978	0.022	0.000	0.000
0.000	0.967	0.033	0.000
1.000	0.000	0.000	0.000
0.217	0.783	0.000	0.000
1.000	0.000	0.000	0.000
0.000	1.000	0.000	0.000
1.000	0.000	0.000	0.000
0.185	0.815	0.000	0.000
0.750	0.000	0.098	0.152

Motif 10

A	C	G	T
0.000	0.000	1.000	0.000
0.339	0.000	0.214	0.446
0.339	0.250	0.125	0.286
0.000	0.000	1.000	0.000
0.500	0.286	0.214	0.000
1.000	0.000	0.000	0.000
0.000	0.000	1.000	0.000
1.000	0.000	0.000	0.000
0.518	0.250	0.232	0.000
0.000	0.000	1.000	0.000

Motif 11

A	C	G	T

0.629	0.000	0.251	0.120
0.619	0.037	0.203	0.141
0.434	0.478	0.030	0.058
0.790	0.088	0.121	0.000
1.000	0.000	0.000	0.000
0.000	0.000	0.000	1.000
0.000	0.000	1.000	0.000
0.000	0.000	1.000	0.000
0.263	0.672	0.012	0.053
0.130	0.000	0.656	0.215

Motif 12

A	C	G	T
0.261	0.108	0.079	0.552
0.015	0.291	0.000	0.695
0.000	0.000	0.000	1.000
0.847	0.000	0.044	0.108
0.000	0.000	1.000	0.000
0.000	0.000	1.000	0.000
0.000	0.000	1.000	0.000
0.000	0.049	0.015	0.936
0.000	0.000	0.000	1.000
0.000	0.128	0.000	0.872

Motif 13

A	C	G	T
0.102	0.085	0.136	0.678
0.017	0.983	0.000	0.000
0.373	0.000	0.000	0.627
0.000	0.847	0.051	0.102
0.000	1.000	0.000	0.000
0.000	0.000	1.000	0.000
0.000	0.000	0.915	0.085
0.085	0.813	0.000	0.102
0.000	0.051	0.949	0.000
0.949	0.000	0.051	0.000
